# Development and pilot testing of Baby-Led Introduction to SolidS - a version of Baby-Led Weaning modified to address concerns about iron deficiency, growth faltering and choking

**DOI:** 10.1186/s12887-015-0422-8

**Published:** 2015-08-26

**Authors:** Sonya L. Cameron, Rachael W. Taylor, Anne-Louise M. Heath

**Affiliations:** Department of Human Nutrition, University of Otago, Dunedin, 9054 New Zealand; Department of Medicine, University of Otago, Dunedin, 9016 New Zealand

**Keywords:** Baby-led weaning, Complementary feeding, Baby-led introduction to solids, Iron intake, Choking, Energy intake

## Abstract

**Background:**

In Baby-Led Weaning (BLW), infants are offered ‘finger’ foods from the start of the complementary feeding period instead of being spoon-fed. Healthcare professionals have expressed concerns about adequacy of iron and energy intake, and about choking, for infants following Baby-Led Weaning.

**Methods:**

We developed a modified version of BLW, Baby-Led Introduction to SolidS (BLISS), to address these concerns. In a 12-week pilot study, families who had chosen to use a BLW approach were assigned to BLISS (*n* = 14) or BLW (*n* = 9). BLISS participants received 2 intervention visits, resources and on-call support. BLW participants received no intervention. Participants were interviewed weekly for 12 weeks and completed a three-day weighed record or three 24-h iron questionnaires.

**Results:**

Compared to the BLW group, the BLISS group were more likely to introduce iron containing foods during the first week of complementary feeding, and to offer more serves per day of iron containing foods at 6 months (2.4 *vs* 0.8 serves/day; *P* = 0.001); and less likely to offer high-choking-risk foods (3.24 *vs* 0.17 serves/day; *P* = 0.027).

**Conclusions:**

This pilot study suggests BLISS may result in higher iron intakes and lower choking risk than unmodified BLW. However, the results need to be confirmed in a large randomised controlled trial.

## Background

Baby-Led Weaning (BLW) is an alternative method for introducing complementary foods to infants [1]. Unlike the traditional method of infant feeding [2, 3], BLW in its purest form does not include any spoon-feeding by an adult. Instead, infants are encouraged to feed themselves family foods from the start of the complementary feeding period [1]. Although anecdotal evidence suggests that many parents in New Zealand, the UK, and Canada are following BLW, healthcare professionals [4] and health governing bodies [5] are unwilling to support BLW as a population recommendation because of concerns related to safety and nutrient sufficiency. In particular, healthcare professionals are concerned that infants following BLW will be at increased risk of inadequate iron and energy intakes, and of choking [4].

Achieving adequate iron intake is problematic for infants worldwide [6, 7]. Current infant feeding recommendations advise parents to offer developmentally appropriate iron-rich foods from the start of the complementary feeding period, particularly if this occurs at the recommended 6 months of age [2, 3, 8–11]. Iron-fortified rice cereal is a popular and convenient food used to increase iron intake in New Zealand [3] as well as other [2, 10] countries, and has been associated with higher iron status [12]. However, because baby-rice cereal has a semi-liquid consistency, and spoon-feeding is not advocated in BLW, this important source of iron is unlikely to be consumed by most infants following BLW.

Healthcare professionals have also suggested that BLW may increase the risk of growth faltering because infants following BLW may not be able to self-feed enough food to meet their energy requirements for growth [4]. The energy density of the foods offered may also be low (e.g., BLW infants may receive only fruits and vegetables as these can be easily prepared as finger foods). To date, no studies have collected detailed dietary data from infants following BLW, although descriptive data from parents who follow BLW suggest that fruits and vegetables are the most commonly offered first foods [4, 13].

Both healthcare professionals and parents have expressed concern about the potential risk of choking when infants follow BLW [4, 14]. In our earlier qualitative study of BLW, 30 % (*n* = 6/20) of mothers reported that their child had choked. One major difficulty with collecting choking data is the ability of parents to differentiate between choking and gagging (which is far more common), making it unlikely that the true rate of choking was this high. However, there does appear to be a lack of knowledge amongst parents about safe and appropriate ‘finger’ foods to offer, with the majority of cases of choking in our qualitative study being associated with consumption of raw apple, an inappropriate food to be offering infants [4].

Given the apparent increase in the popularity of BLW amongst families, a number of possible risks, including concerns about iron and energy intake, as well as the potential risk of choking, need to be addressed. Therefore, the aims of this study were: first, to develop a modified version of BLW, called Baby-Led Introduction to SolidS (BLISS), which encourages parents to introduce complementary foods using a baby-led approach, but which has been modified to address concerns about iron, energy and choking; and second, to conduct a pilot study to determine the extent to which parents following this modified baby-led approach offer foods that are likely to increase iron and energy intake, and to lower choking risk.

## Methods

### Development of Baby-Led Introduction to SolidS

Baby-Led Introduction to SolidS (BLISS) was developed by the authors with the assistance of a paediatrician and a paediatric speech-language therapist. It is a version of BLW modified to address the three primary concerns of healthcare professionals, parents and the authors [4, 15]:

1. Increased risk of choking, because finger foods are offered at a younger age than has traditionally been advised, the infant does not have the opportunity to ‘learn’ to eat finger foods as they would if they began with purées.
2. Increased risk of low iron status, because the iron-fortified foods that are often relied on to provide much of the iron needed in the complementary feeding period (e.g., ‘baby rice’) are designed for spoon-feeding.
3. Increased risk of growth faltering, because infants may not have the necessary skills to pick up food or the stamina to consume enough food to match their energy needs for appropriate growth, and because easy-to-hold, but low energy, fruit and vegetables may form the basis of the infant’s diet.

The essential characteristics of BLISS are:

1. Offer foods that the infant can pick up and feed themselves (i.e., follow a BLW approach)
2. Offer one high-iron food at each meal.
3. Offer one high-energy food at each meal.
4. Offer food prepared in a way that is suitable for the infant’s developmental age to reduce the risk of choking, and avoid offering foods listed as high-choking-risk foods.

### Development of resources

The primary vehicle for delivering the BLISS education, advice and target behaviours was a collection of booklets that were discussed at individual meetings with parents at 5.5 and 7 months of age. As the resources were intended to suit the general public, special consideration was given to the language used and images included. The language was ‘everyday’ and culturally appropriate. The terms used reflected those commonly used by New Zealand parents (e.g., complementary foods were referred to as ‘solids’) and the images resembled New Zealand children and families. Resource presentation was completed in collaboration with a graphic designer and was intended to be eye-catching and engaging. The resources outlined specific recommendations, for example infants should receive one high-iron food at each meal, and offered practical advice (e.g., high-iron recipes) on how to achieve the recommendation. In addition, to add authenticity to the resources, supporting quotes and anecdotes from parents who had previously used BLW were included. At 5.5 months, the resources covered the topics: what to expect from a baby-led approach to solids; safety when starting solids; what, when and how to offer first foods (and recipes); offering a high-iron, a high-energy, and an ‘easy’ to pick up and eat food at each meal (with specific age-appropriate examples); and how to tell whether an infant is hungry or full. At 7 months, the resources covered the topics: suggestions for more challenging textures and tastes (and recipes); and offering a high-iron, a high-energy, and an ‘easy’ to pick up and eat food at each meal (with specific age-appropriate examples). The messages regarding increasing iron intake, reducing the risk of growth faltering, and preventing choking are listed in Table 1.

**Table 1:** BLISS recommendations developed to address low iron and energy intake, and the potential risk of choking

Aim of recommendation	Specific recommendations for parents	Recommendation guided by
Increase the intake of high-iron foods	1). Encouraged to offer a high-iron food at each meal.	Nutritionist with expertise in iron nutrition (A-LH)
	2). Provided with ideas for increasing the iron content of foods (e.g., including iron-fortified infant rice cereal in baking).	
	3). Provided with recipes and food ideas for iron-containing foods (including red meat which is high in total iron, haem iron, and the “meat/fish/poultry” factor that enhances non-haem iron absorption).	
	4). Advised to begin complementary feeding at 6 months of age (i.e., not to delay beyond 180 days).	
Reduce the risk of growth faltering as a result of low energy from self-feeding	1). Encouraged to offer a variety of foods, including at least one high-energy food at each meal.	Paediatric health professionals
	2). Provided with food ideas and recipes that were high in energy and could be easily self-fed by the infant.	
	3). Encouraged to practice responsive feeding, ensuring that: the feeding environment is pleasant with few distractions (e.g., no television), caregivers pay attention to the infant’s hunger and satiety cues, and that caregivers respond to the infant promptly and supportively.	
	4). Encouraged to offer ‘easy’ foods and more frequent milk feeds when their child was ill and during recovery.	
Reduce the risk of choking	1). Advised to test foods before they are offered to the infant to make sure they are soft enough to mash with the tongue on the roof of the mouth.	Paediatric speech-language therapist
	2). Provided with a list of specific foods to avoid (e.g., raw apple).	
	3). Advised to also avoid: foods that form a crumb in the mouth, hard foods, small foods, and circular (coin) shaped foods.	
	4). Educated on safety around eating including how to differentiate between gagging and choking, and what to do if choking occurs.	

### Development of recipes

A range of high-iron recipes and high-energy recipes was developed in the Department of Human Nutrition Bristol-Myers Squibb Metabolic Kitchen (University of Otago, Dunedin, New Zealand) and tested for consistency (could be picked up without falling apart) and palatability as family foods (as determined by a convenience sample of *n* = 4 adults). A preliminary food list was compiled from the high-iron and high-energy foods developed in the recipe testing, as well as foods that are currently offered to New Zealand six-month-old infants [3]. Any foods that were deemed by the paediatric speech-language therapist to present a high risk of choking were excluded. The BLISS high-iron recipes included red meat, liver, iron-fortified infant cereal, or legumes, and contained an average of 2.1 mg of iron per 100 g (1.3 mg of iron per 100 kcal). The high-energy recipes provided more than 1.5 kcal/g (i.e., 6.3 kJ/g). Recipes that were safe, palatable, feasible (inexpensive and convenient), and had a consistency that did not fall apart when held were included in the resources.

### Pre-testing of resources

The resources were first tested in a convenience sample of six parents for readability, acceptability and comprehension. These six parents had similar age, level of education and parity to those in the Pilot study, and a similar proportion were New Zealand European. On the basis of the feedback from this testing, a number of statements were reworded to improve clarity, and additional recipes were added. The resources then underwent expert review. Six experts from the fields of paediatrics, nutrition for young children, and first aid reviewed the resources. As a result, additional first aid and safety information was added, and a number of statements in the ‘Safety around starting food’ resource were reworded for clarity.

### Pilot study

#### Participants and recruitment

Families with a child aged five months were recruited using an advertisement in the Dunedin Star Newspaper. The Star is a free weekly newspaper which is delivered to more than 43,500 homes throughout the urban area of Dunedin city and its environs. The advertisement stated that we were seeking participants with a child up to five months of age who were intending to use a baby-led approach to introduce ‘solids’ to their infant. At first contact, a brief overview of the study was given to parents and an information sheet about the study was sent to their home address or email. Three days after the information sheet was sent, parents were telephoned for follow-up. Parents who wanted to participate in the study were sent a consent form to complete.

Parents were not eligible if their child was born prematurely (less than 34 weeks gestation), had developmental delay diagnosed by a health professional, or had feeding or swallowing difficulties. Participants chose whether they wanted to be in the BLW or BLISS group, except one participant who was not eligible for the BLISS group because they were enrolled in another study measuring infant feeding outcomes that may have been influenced had they modified their behaviour as a result of participating in this pilot study.

On completion of the study all participants received a supermarket voucher to the value of $20. The study was approved by the Human Ethics Committee of the University of Otago, Dunedin, New Zealand, and all participants provided written informed consent.

### Intervention

Participants in the BLISS group received resources at two individual home visits when the infant was 5.5 months and 7 months of age. The first set of resources was delivered to parents when the baby was 5.5 months of age to allow a 2-week familiarisation time before starting BLISS when their baby turned 6 months of age. Participants were encouraged to start offering complementary foods as soon as their infant turned 6 months of age (i.e., at 180 days) both to discourage earlier introduction of solid foods (which we judged to be unsafe because of the risk of choking), and to discourage later introduction (which we considered would increase the risk of iron deficiency [8]). Participants were advised to offer puréed food if they decided to start complementary foods before 6 months of age, then to start BLISS at 6 months.

Additional resources were delivered at seven months of age, on the advice of the paediatric speech-language therapist that children are developmentally more advanced and ready to manage new textures and shapes of food at this age. The home visits were based on the resources, with delivery tailored to individual participants, and typically lasted one hour. In addition, individualised advice and support from the research staff was available on request throughout the study (this was accessed by one participant who asked for advice on how to encourage her mother-in-law to accept a baby-led approach to infant feeding).

Participants in the BLW group were not given any feeding protocol to follow. Instead, they were asked to follow BLW as they had intended at baseline, and to be available for an interview each week for 12 weeks from 6 months of age.

All participants were able to access the standard “Well Child” care that is provided to all New Zealand families free of charge from birth until their child is five years of age (http://www.health.govt.nz/publication/well-child-tamariki-ora-national-schedule-2013).

### Data collection

All participants were asked to complete a structured 30-min telephone interview weekly (‘weekly interview’) for 12 weeks from 6 to 9 months of age. Demographic information was collected during the baseline interview. Data on the iron content of the complementary foods offered were collected in three ways: all participants completed the weekly interview, a subsample of ten participants (*n* = 5 from BLISS, *n* = 5 from BLW) whose child was aged 6 months agreed to complete a 24-h iron questionnaire on three non-consecutive days (‘3-day iron questionnaire’), and a different subsample of eight participants (*n* = 4 from BLISS, *n* = 4 from BLW) whose child was aged 6 months agreed to complete a weighed diet record on three non-consecutive days (‘3-day weighed record’). Data on the energy content of the complementary foods offered, and on the high-choking-risk foods offered were collected in two ways: from the weekly interview, and from the 3-day weighed record.

The interview schedule used for the weekly interviews with all participants is shown in Table 2. The data collected during the weekly interviews were used to determine: a) adherence to a baby-led approach to complementary feeding (the percentage of self-feeding, shared family meals, and food that was family food); b) the number of different (i.e., variety) of iron containing foods, high-energy foods, and high-choking-risk foods that had been offered; c) whether gagging or choking had occurred and which foods were responsible; d) the number of meals eaten per day.

**Table 2:** Weekly interview schedule

1. What foods has your baby had this week?
2. Have you tried any new foods this week?
3. What percentage of the foods eaten were from the family meal?
4. (a). Is [baby’s name] eating at the same time as the rest of the family?
(b). If yes, how often is [baby’s name] eating at the same time as the rest of the family?
5. How often is [baby’s name] having solids each day?
6. What percentage of [baby’s name] total food did she/he feed him/herself?
7. What percentage of [baby’s name] total food was he/she spoon-fed?
8. (a). Has [baby’s name] gagged this week?
(b). If yes, on what?
(c). How did you know she was gagging?
(d). Was it food she/he fed him/herself?
(e). What did you do?
9. (a). Has [baby’s name] choked this week?
(b). If yes, on what?
(c). How did you know she was choking?
(d). Was it food she/he fed him/herself?
(e). What did you do?

The 3-day iron questionnaire was administered when infants were between 6.5 and 7 months of age. On three different days, the participants were asked to recall how often, in the previous 24 h, they had offered foods from a list of iron containing foods developed by the authors (Table 3). These data were used to determine the number of serves of iron containing foods offered per day. This questionnaire was introduced part way through the study and all ten families with an infant aged 6.5–7 months at that time were asked to complete the 3-day iron questionnaire.

**Table 3:** Descriptive food lists developed to compare BLW and BLISS eating patterns

Foods classified as iron containing foods
Beef
Chicken
Fish
Ham
Lamb
Bacon
Liver (including pâté)
Luncheon sausage or other sausage
Pork
Salami
“Saveloys” or “cheerios” (processed meat sausages)
Iron-fortified infant rice cereal
Baked beans
Lentils
Hummus
Chickpeas (other than hummus)
Foods classified as high-energy foods
All foods except *most* fruit and vegetables, plain rice crackers, or clear soups were classified as high-energy foods.
Fruits classified as high energy: Avocado and banana
Vegetables classified as high-energy: Pumpkin, potato and kumara (sweet potato).
Foods classified as high-choking-risk foods
Raw vegetables (e.g., carrot, celery, salad leaves)
Raw apple
Rice crackers, potato crisps, corn chips
Whole nuts
Dried fruit (e.g., raisins, cranberries)
Cherries, grapes, berries, cherry tomatoes
Peas, corn
Lollies (i.e., sweets or candy)
“Saveloys”, hotdogs (processed meat sausages)
Other hard food (i.e., foods that could not be squashed against the roof of the mouth with the tongue)

The 3-day weighed record was delivered to participants in their home when their infant was 6 months of age. The participants were given verbal and written instructions on how to collect the record and were given the opportunity to ask questions. The record was collected using dietary scales accurate to within 1 g (Salter Electronic, Salter Housewares Ltd, Tonbridge, UK) on three non-consecutive days, including two weekdays and a weekend day, over a week. The dietary data, excluding breast milk and infant formula intake, were entered into the dietary analysis programme Kai-culator (Department of Human Nutrition, University of Otago, Dunedin, New Zealand) which accesses the New Zealand food composition database FOODfiles (Plant & Food Research, Palmerston North, New Zealand), and analysed to determine the energy (kJ/day) and iron (mg/day) content of the complementary foods offered. In addition, the number of serves per day of iron containing , high-energy and high-choking-risk foods offered was calculated manually. The last four BLW families recruited into the study, and four BLISS families with babies of a similar age at that time, were asked to complete the 3-day weighed record when their infant was 6 ½–7 months of age). The participants who completed the 3-day weighed record were, therefore, not the same participants who had completed the 3-day iron questionnaire.

### Development of descriptive food lists

Three descriptive food lists were developed for: 1) iron containing foods, 2) high-energy foods, and 3) high-choking-risk foods. These descriptive food lists were used to develop the 3-day iron questionnaire, and to interpret data from the weekly interview and 3-day weighed record. The criteria for inclusion in the lists were based on guidance from nutrition and paediatric experts. The lists were designed to describe foods that were being offered to the infants – they were not used to recommend foods. For example, bacon was included in the descriptive food list for iron containing foods, even though it is not an appropriate food for this age group because of its high sodium content, because it would have contributed to iron intake if it had been consumed.

Foods included in the iron containing descriptive food list were: meat, chicken, fish and liver (because of their iron content; the presence of well-absorbed haem iron; and the presence of the ‘meat/fish/poultry factor’, a powerful enhancer of iron absorption [16]), iron-fortified infant cereal (the only fortified food on the New Zealand market with a high enough level of iron (2.5–4 mg/100 g) to make an appreciable difference to iron intake in the small portion size consumed by infants), and legumes that would be expected to be eaten by New Zealand vegetarian infants (because of their high iron content) (see Table 3).

Foods included in the High-energy descriptive food list were foods providing greater than 1.5 kcal/g (see Table 3). This criterion was adopted from earlier studies [17, 18] on the appropriate energy density for complementary foods for young children.

Foods in the High-choking-risk descriptive food list were specific foods that the paediatric speech-language therapist had advised against offering (which had been included in the BLISS safety resource as foods to avoid), and any additional foods that were hard, small, coin-shaped, or dry and likely to crumble in the mouth (see Table 3).

### Statistical analysis

All analyses were conducted using Stata^™ ^ version 12 [19]. For all analyses and reporting, the term ‘6 months’ refers to the month from 6 months 0 weeks of age to the end of 6 months 3 weeks of age. The terms ‘7 months’ and ‘8 months’ should be interpreted similarly. Mothers were assigned to mutually exclusive ethnic groups using the 2006 New Zealand National Census question [20]. Participants who nominated two or more ethnic groups were assigned to a single group using the prioritisation system recommended by Statistics New Zealand, with the order of priority being (from highest to lowest): Māori, Pacific, Asian, Other, New Zealand European [20]. Differences in proportions of self-feeding, family meals shared, and family foods eaten were compared between the two groups (BLISS *vs* BLW) at each time period: 6 months, 7 months, and 8 months. Fisher’s Exact test (two-tail) and Pearson chi-squared were used to identify differences in demographic variables (maternal age, ethnicity, education, parity, and employment status) and feeding variables (number of serves per day of iron containing foods, high-energy foods and low-energy foods, high-choking-risk foods; variety of iron containing foods, high-energy foods, high-choking-risk foods; number of meals per day, and choking incidents). Student’s paired *t*-test was used to test for significant differences between continuous variables including the amount of energy (kJ/day) and iron (mg/day) offered from complementary foods from the three-day diet records. A *P*-value of <0.05 was considered to indicate statistical significance.

## Results

### Participant characteristics

Twenty-five families who had a child aged 5 months and who were intending to use a baby-led approach to introduce complementary foods to their infant were examined for eligibility. Two families were excluded from the study before consent was obtained (because the infant was born before 34 weeks gestation, or had swallowing difficulties self-reported by the mother). The final number of participants was 23 (*n* = 14 BLISS, *n* = 9 BLW). The mean (SD) age of the participants was 31.2 (3.5) years. More than half of the sample had a university degree (65 %, *n* = 15/23), were New Zealand European (74 %, *n* = 17/23), were primiparous mothers (70 %, *n* = 16/23), and in paid employment (  74 %, n = 17/23). There were no significant differences between the groups for these demographic variables (maternal age *P* = 0.674; maternal education *P* = 1.000; maternal ethnicity *P* = 0.200; parity *P* = 0.052 and maternal employment status *P* = 0.475).

### Adherence to Baby-Led Introduction to SolidS

Feeding behaviours, as described in the weekly interview, are summarised in Table 4. There were no differences between the BLISS and BLW groups in the measures of adherence to the baby-led approach (proportion of self-feeding, family foods eaten, family meals shared with the child) at any of the ages (6 months, 7 months, or 8 months).

**Table 4:** Feeding behaviours of participants in the BLISS and BLW groups (data from weekly interviews)^1^

		6 months (%)	*P*-value^*^	7 months (%)	*P*-value^*^	8 months (%)	*P*-value^*^
Proportion of food that the infant self-fed	*BLW*	94.4	0.736	88.1	0.816	89.0	0.738
	*BLISS*	90.5		91.5		93.5	
Proportion of food that was family food^2^	*BLW*	84.7	0.469	97.2	0.816	97.2	0.900
	*BLISS*	93.8		94.6		96.4	
Proportion of meals that were shared with the family^3^	*BLW*	100.0	0.392	100.0	0.672	98.6	0.649
	*BLISS*	90.6		96.4		93.8	
Number of meals per day^4^	*BLW*	2.3	0.599	2.9	0.731	3.4	0.101
	*BLISS*	2.0		2.7		3.5	

^1^BLW group *n* = 9; BLISS group *n* = 14

^2^Eating the same food as the family but not necessarily eaten at the same time

^3^Eating at the same time as the family but not necessarily eating the same food

^4^Meals had at least one “solid” food – meals comprising only breast milk or infant formula were not included

^*^ *P*-value compares BLW and BLISS groups. Bold indicates significance (P < 0.05)

### Iron

The amount of iron offered from complementary foods (mg/day) (according to the 3-day diet records) was not statistically significantly different between the BLISS (4.9 mg/day) and BLW (2.2 mg/day) subsamples (*P* = 0.110). However, grams of red meat offered per day was significantly higher in the BLISS (20.1 g/day) compared to the BLW group (3.2 g/day) (*P* = 0.014). In addition, a wider variety of iron containing foods was offered in the BLISS group than the BLW group at all three time periods (Table 5), according to the weekly interviews. A greater number in the BLISS group introduced iron containing foods to their child when they first started complementary foods (i.e., during week one) compared to the BLW group (78.6 vs. 22.3 %; *P* = 0.007). Data from the 3-day iron questionnaire (*n* = 10) and the diet records (*n* = 8) confirmed that BLISS participants offered more serves per day of iron-containing foods at 6 months (2.4 vs. 0.8 serves/ day) than BLW participants (*P* = 0.001).

**Table 5:** Mean (SD) number of foods offered on at least one occasion per week by participants in the BLISS and BLW groups (data from weekly interviews)^1,2^

		6 months	*P*-value^*^	7 months	*P*-value^*^	8 months	*P*-value^*^
High-energy foods	BLW	17.8 (8.8)	**0.049**	30.1 (9.2)	**0.001**	32.3 (11.1)	**0.013**
	BLISS	26.7 (10.6)		47.2 (10.5)		47.9 (14.6)	
Iron containing foods	BLW	4.9 (2.8)	**0.016**	7.8 (4.0)	**0.002**	9.4 (4.2)	**0.001**
	BLISS	10.6 (6.1)		17.6 (7.5)		19.7 (6.6)	
High-choking-risk foods	BLW	3.4 (3.0)	**0.026**	4.4 (4.6)	0.138	4.3 (3.8)	**0.029**
	BLISS	1.1 (1.6)		2.2 (2.3)		1.4 (2.1)	

^1^ BLW group *n* = 9; BLISS group *n* = 14

^2^ These data are not a count of the number of serves of food offered, but of the number of *different* foods offered so are an indicator of food variety rather than quantity

^*^ *P*-value compares BLW and BLISS groups. Bold indicates significance (P < 0.05)

### Energy

The amount of energy offered from complementary foods (kJ/day) (according to the 3-day diet records) was not statistically significantly different between the BLISS (2228 kJ/day) and BLW (1862 kJ/day) subsamples (*P* = 0.494). Similarly, according to the diet records, there was no difference between the BLISS and BLW subsamples for the mean number of serves of high-energy foods offered per day at 6 months (Table 6), or the mean number of low-energy foods (fruit and vegetables) at 6 months. However, a wider variety of high-energy foods was offered, on at least one occasion, by the BLISS group compared to the BLW group at 6, 7, and 8 months (Table 5). The mean number of meals eaten by the infants in the BLISS and BLW group at 6, 7, and 8 months was not significantly different (Table 5), with an average of 2.8 meals/day.

**Table 6:** Mean (SD) serves offered per day by participants in the BLW and BLISS groups at 6 months of age (data from 3-day weighed records)

	BLW (*n* = 4)	BLISS (*n* = 4)	*P*-value^*^
High-iron foods	0.73 (0.5)	2.40 (0.5)	**0.001**
High-energy foods	5.92 (1.7)	6.32 (3.3)	0.836
Low-energy foods (fruits and vegetables)	3.82 (1.7)	5.82 (1.0)	0.229
High-choking-risk foods	3.24 (1.6)	0.17 (0.2)	**0.027**

^*^ *P*-value compares BLW and BLISS groups. Bold indicates significance (P < 0.05)

### Choking

The incidence of choking reported in the weekly interviews was not different between the groups: two choking incidents were reported in the BLISS group, and one in the BLW group. Raw apple and grapes were the foods reported to have caused the choking. All choking incidents were dealt with at home and did not require medical intervention. There was no difference in the proportion reporting a gagging incident according to the weekly interview data at 6 (BLW 0.53 vs. BLISS 0.54), 7 (BLW 0.42 vs. BLISS 0.14) or 8 (BLW 0.14 vs. BLISS 0.07) months of age between the BLW and BLISS groups (*P* > 0.05).

Data from the weekly interviews do, however, show that the BLISS infants were significantly less likely to be offered high-choking-risk foods compared to the BLW infants at 6 months and 8 months, although this was not significantly different at 7 months (Table 5). The 3-day weighed record data at 6 months confirms that the number of serves of high-choking-risk foods offered per day was significantly lower in BLISS participants than in BLW participants (*P* = 0.027) (Table 6).

## Discussion

Overall, the Baby-Led Introduction to SolidS approach to complementary feeding was accepted and implemented by the parents in this pilot study. The BLISS approach resulted in a greater number of iron containing foods and a lower number of high-choking-risk foods being offered to infants at 6 months of age compared to the BLW approach. Although there was no difference observed in the number of serves of high-energy foods offered, a wider range of high-energy foods was offered to those in the BLISS compared with the BLW group.

Adherence (the proportion of self-feeding, family foods eaten, family meals shared with the child) to a baby-led approach to complementary feeding was not different between the BLISS and BLW groups. This may be due to many of the participants belonging to parenting groups that offered BLW support. Furthermore, both groups were recruited on the basis that they felt confident to follow BLW independently from the study. Brown and Lee [13, 21] have shown in a larger sample that when participants are recruited from populations that define themselves as following BLW, they adhere strongly to BLW principles (i.e., less than 10 % spoon-feeding and 10 % purées, having family meals together, and offering family foods), although this is certainly not the case for all parents who consider themselves to be following BLW [15].

Food-based approaches are recommended as strategies to prevent iron deficiency in populations where mild deficiency exists [22], as it does in New Zealand infants [7]. The BLISS intervention promoted new recipes that incorporated iron-fortified rice cereal as well as encouraging consumption of high-iron foods such as beef. Previous studies have shown that foods containing iron-fortified cereal are acceptable to infants, and have demonstrated that these foods can improve the iron status of infants [12] as much as medicinal iron [23, 24] in non-anaemic populations. In addition, interventions promoting red meat intake have also been shown to be feasible, affordable and efficacious in improving infants’ and toddlers’ iron intake and status [25–27]. The BLISS intervention increased the number of serves of iron containing foods offered per day. In addition, BLISS also resulted in a wider range of iron containing foods being offered from 6 to 9 months of age. Although there was no statistically significant difference in the amount of iron offered from complementary foods by the BLISS (4.9 mg/day) and BLW (2.2 mg/day) participants who completed the diet records, the sample size was very small (4 in each group), and the BLISS group did offer a substantially greater amount of red meat (20.1 g/day) than the BLW group (3.2 g/day) (*P* = 0.014). Meat, in particular red meat, is a good source of bioavailable haem iron [28] which, unlike non-haem iron, is little affected by ingestion of inhibitory dietary components such as phytate [29]. In addition, the presence of meat in the diet also enhances the absorption of non-haem iron [26, 30]. Therefore, a higher intake of red meat in the BLISS group is promising in terms of improving the absorption of iron from the diet, and perhaps resulting in better iron status. However, these results need to be corroborated in a large sample that includes measures of biochemical iron status. Due to the extremely high dietary iron requirement of infants at 6 months of age, the New Zealand Ministry of Health [3] and the World Health Organization (WHO) [8] recommend that infants begin high-iron foods immediately they start complementary foods. In the current pilot study we observed that a substantially greater number of BLISS compared to BLW infants (78.6 vs. 22.3 %) were offered iron-containing foods in the first week of starting complementary foods. However, it is of concern that none of the 8 infants for whom diet record data were available were achieving the WHO recommendation for iron intake from complementary foods of 10.8 mg/day (assuming medium bioavailability and average breast milk intake), even using these data which measured the amount offered so overestimate the amount consumed [31]. Both the BLISS and the BLW groups were thus potentially at increased risk of suboptimal iron status.

The large increase in the amount of red meat offered is likely to have increased protein, as well as iron, intake. It is therefore important to investigate protein intake in a larger randomized controlled trial in which infants following BLISS are compared with control infants who are being fed using more traditional methods. There are a number of possible effects of high protein intake in infants [32], including increased risk of overweight and obesity in later life. One of the few studies that have investigated the effects of higher protein intakes around the time when complementary foods are introduced reported greater weight gain between 5 and 10 months of age amongst infants with protein intakes ≥16 % of energy [33], and other studies have suggested that higher protein intakes in older infants may be associated with higher BMI [34] or body size [35] at 7 to 10 years of age. The infants in the BLISS group in the current pilot study were being offered complementary foods providing 14.7 % of energy as protein (compared to 10.8 % for the BLW group). This is similar to the intake reported for Danish 9 month old infants (13–14 %; [35]), but would be lower once breast milk or infant formula intake was included, and once offered but uneaten food was taken into account. Unfortunately, the Institute of Medicine does not provide an Acceptable Macronutrient Distribution Range (AMDR) for this age group, but the AMDR for infants 1–3 years of age is very wide and includes our reported value (5–20 %) [36]. The results of the current pilot study suggest that the majority of infants following BLISS are unlikely to have excessive protein intakes, however, randomised controlled trial data are required to determine whether some individuals may have inappropriately high intakes.

To address the concern that the majority of foods offered when a baby-led approach to complementary feeding is followed may be low-energy foods, BLISS participants were encouraged to offer at least one high-energy food at each meal. Participants were educated about what constitutes a high-energy food and provided with recipes and high-energy food ideas. However, both groups offered similar amounts of high-energy foods. High-energy food was defined in the current pilot as providing greater than 1.5 kcal/g [17]. This figure was chosen as it has previously been shown to be a minimum energy density for foods offered to healthy breastfed infants [17]. Although this classification was from a Bangladeshi population and may therefore not be ideal for New Zealand infants, it was the only available cut-off for infants during the introduction of complementary foods. This classification resulted in all foods except the majority of fruit and vegetables, plain rice crackers, and soup broth being classified as high energy foods and this may have made it more difficult to detect differences in the energy content of the foods offered. However, we found there was no difference between the BLISS and BLW groups for the mean amount of energy offered from complementary foods or the number of serves of high-energy foods offered to the infants in the BLISS and BLW groups (data from the 3-day weighed record). It is important to note, though, that the infants in the BLISS group were receiving a greater variety of high-energy foods (data from the weekly interviews). It is possible that the BLISS resources and recipes enabled the parents to expand their food repertoire, hence increasing the BLISS infants’ dietary variety.

All of the infants who provided diet record data were being offered amounts of food that exceeded (by 403 to 1999 kJ/day) the WHO recommendation for energy to be provided by complementary food at 6 months of age (838 kJ/day) [37]. In light of this, it is interesting to review a concern raised by healthcare professionals that BLW infants are likely to be offered only fruit and vegetables and thus have inadequate energy intakes [4]. It is important to note that the current pilot study measured the amount offered rather than the amount the infants consumed, so this will be an over-estimate of intake. The energy intake and subsequent growth of infants following a baby-led approach warrants further investigation to determine the extent to which a baby-led approach meets or exceeds the energy needs of infants, and how this compares with the energy intakes and growth of spoon-fed infants.

In the current pilot study there was no significant difference in the rates of choking between infants following unmodified BLW, and those following BLISS which had been modified to decrease the risk of choking. However, the reported rates over the 12-week study were low in both groups (*n* = 3 incidents reported in total). The participants who did report a choking incident noted that the foods that had caused the incident were raw apple (*n* = 2) and raw grapes (*n* = 1), both of which were specifically advised against in the BLISS resources. In our previous work [4], raw apple was also reported as a choking hazard. Furthermore, apple and grape have been associated with fatal choking in young children [38]. These findings support the exclusion of raw apple from infants’ diets [3]. The BLISS group were, however, offering substantially fewer high-choking-risk foods than the BLW group (0.17 foods per day compared with 3.2 foods per day), which would be expected to decrease their choking risk at a population level.

The strengths of this pilot study are the involvement of multiple experts in the development of the BLISS approach, the pilot study’s prospective nature, and the weekly follow-up, which reduced the risk of recall bias. There are, however, some methodological limitations of this pilot study. First, there was no group of conventional feeders (i.e., parents spoon-feeding purées) for comparison. Second, the participants were not randomly assigned to their group. At the time that the pilot study was being designed, concerns had been expressed about whether a baby-led approach to infant feeding may increase the risk of iron deficiency, growth faltering or choking, both by health professionals [4], and by the research team themselves (hence our modification of BLW when designing the BLISS approach). Moreover, there had been no randomised controlled trials in which participants had been asked to follow a baby-led approach to infant feeding, and therefore no studies that had demonstrated its safety. It was not, therefore, considered ethical to recruit participants unless they were already planning to follow BLW. Similarly, in the absence of evidence that BLISS was safe, we allowed the participants to choose which approach they would use. Although this meant that parents who felt confident about independently following BLW became the BLW group, whereas others who felt they needed extra support became the BLISS group, the groups adhered to a baby-led approach to the same extent, and their age, education, ethnicity, parity, and employment status did not differ. It is still possible, however, that there were other unmeasured differences that led to those in the BLISS group responding to the BLISS intervention differently to the way that those in the BLW group may have responded. Third, some followers of BLW do not wait until 6 months of age before introducing whole foods, as the participants in this pilot were required to, so their outcomes may differ to those seen in this study. Fourth, we measured parental behaviour, i.e., food offerings, rather than infant intake per se. Fifth, in this pilot study we did not measure iron status or growth, which are the ultimate indicators of the adequacy of iron and energy intakes. Lastly, and perhaps most importantly, the sample size was very small (as befitting a pilot study) and some data (e.g., weighed records) were collected from only a subsample of participants. Thus our results should be interpreted with caution and require confirmation from a larger, adequately powered randomised controlled trial that investigates intake of a wide range of nutrients, and includes measurements of iron status and growth. This pilot study has, however, demonstrated that a BLISS approach to infant feeding is feasible, and that use of the resources developed and tested in this pilot study is likely to result in behaviour change in participants in a larger randomised controlled trial.

## Conclusions

The BLISS intervention was able to reduce the offering of high-choking-risk foods and to increase the offerings and variety of iron containing foods. As food-related choking in children and suboptimal iron status have been suggested to be particular concerns when a baby-led approach to complementary feeding is followed [4, 15], and choking and iron deficiency are already two major health risks for New Zealand infants [7, 38, 39], these results warrant further investigation. This pilot study was preparatory work for a randomised controlled trial where the effectiveness of BLISS can be determined in a large sample in which accurate measures of nutrient and energy intake, and choking, are collected alongside biochemical iron status and growth data – the Baby-Led Introduction to SolidS study.

## Abbreviations

AMDR: Acceptable Macronutrient Distribution Range
BLISS: Baby-Led Introduction to SolidS
BLW: Baby-Led Weaning
UK: United Kingdom
WHO: World Health Organization
